# Adapting TeamSTEPPS for school mental health teams: a pilot study

**DOI:** 10.1186/s40814-019-0529-z

**Published:** 2019-12-17

**Authors:** Courtney Benjamin Wolk, Rebecca E. Stewart, Peter Cronholm, Ricardo Eiraldi, Eduardo Salas, David S. Mandell

**Affiliations:** 1 Center for Mental Health Policy and Services Research, Perelman School of Medicine, University of Pennsylvania, 3535 Market Street, Floor 3, Philadelphia, PA 19104 USA; 2 Department of Family Medicine and Community Health and Center for Public Health Initiatives, University of Pennsylvania, 51 North 39th Street, 6th Floor Mutch Bld, Philadelphia, PA 19104 USA; 3 Division of Developmental and Behavioral Pediatrics and Department of Psychiatry, Perelman School of Medicine, University of Pennsylvania, 2716 South Street, 8th floor, Philadelphia, PA 19146 USA; 4 Department of Psychological Sciences, Rice University, P.O. Box 1892, Houston, TX 77251 USA

**Keywords:** Teams, teamwork, TeamSTEPPS, School mental health, Evidence-based practice

## Abstract

**Background:**

School mental health care often is provided by teams contracted from community mental health agencies. The team members that provide this care, however, do not typically receive training in how to work effectively in a team-based context. Team Strategies and Tools to Enhance Performance and Patient Safety (TeamSTEPPS) provides a promising, evidence-based strategy for improving communication and climate in school-based teams.

**Methods:**

In collaboration with stakeholders, we adapted and piloted TeamSTEPPS for use with school mental health teams. Teams in six schools were randomized to receive the adapted TeamSTEPPS approach or usual supports. The main outcomes of interest were feasibility and acceptability of the adapted TeamSTEPPS strategy.

**Results:**

Results indicated that team member burnout was significantly higher at follow-up than pretreatment for both control and intervention teams. TeamSTEPPS was feasible and acceptable to implement, and leadership emerged as an important facilitator. Barriers to implementation success included staff turnover, lack of resources, and challenges in the school mental health team relationship. Additional supports to implement TeamSTEPPS were suggested, including ongoing consultation and booster training to address high staff turnover.

**Conclusions:**

Results suggest that TeamSTEPPS is promising for school mental health teams but additional modifications are likely needed.

## Background

Children today obtain more mental health services through schools than through any other public system [1]; however, school-based mental health care often is in short supply and of poor quality [2]. Clinician training often is the primary strategy to increase use of evidence-based mental health practices (EBPs) in schools and other community settings; however, many school service providers who have received training in EBPs do not use them more than they use non-evidence-based interventions [3], and there is growing consensus that training is necessary but not sufficient to change practice [4]. Most training strategies neglect the critical role of the organizational context, which likely affects implementation of EBPs [5].

In Philadelphia, most school-based mental health care is provided by teams contracted from community mental health agencies, a common model of school mental health service provision [6]. These teams include clinicians as well as individuals who perform in-class support and case management activities. This care can include individual and group therapy, support in the classroom, and crisis management. In an effort to improve school mental health services in Philadelphia, the Department of Behavioral Health and Intellectual disAbility Services has initiated extensive training and consultation in the broad application of cognitive-behavioral therapy (CBT) [7]. While this initiative represents an important step in advancing quality of care, additional implementation strategies that address contextual factors other than clinician skill may be needed to optimize services [8]. For example, clinicians may benefit from concrete tools for transferring knowledge and skill acquired in CBT trainings to other members of the care team (who interact with youth but are not trained clinicians), supporting best practices across team members, and managing interpersonal and organizational challenges that may arise when delivering treatment as part of a team.

Team processes have been shown to affect clinical performance [9] and team training interventions improve patient outcomes [10, 11]. While team improvement strategies [12] have been applied to medical teams [13], this has yet to be extended to mental health teams. One particular team training intervention, Team Strategies and Tools to Enhance Performance and Patient Safety (TeamSTEPPS [14, 15];), has been widely used in health care settings with encouraging outcomes [16, 17]. TeamSTEPPS improves team skills in leadership, situation monitoring, mutual support, and communication [18]. It has been associated with improved teamwork and patient outcomes [16, 19], and reduced provider burnout [20] and turnover [21].

A team approach like TeamSTEPPS could provide a cost-effective strategy for improving quality and effectiveness of student mental health services both by improving team member experience, which has the potential to reduce professional burnout, as well as by improving clinical care through better team communication and skill transfer. We adapted TeamSTEPPS for school mental health teams in collaboration with an advisory board of key stakeholders, including school mental health team leaders, clinicians, and in-class support staff. Next, we pilot tested the adapted TeamSTEPPS, examined feasibility and acceptability, and the impact of the approach on team skills and behavior. We hypothesized that TeamSTEPPS would be feasible and acceptable and that it would be associated with improved team skills and behavior. We also explored the impact of TeamSTEPPS on provider burnout and the association between perceptions of TeamSTEPPS and staff turnover.

## Methods

The TeamSTEPPS curriculum consists of an introduction and four didactic modules targeting the following competencies: team structure, leadership, situation monitoring, mutual support, and communication [14, 15, 18]. The content emphasizes defining team skills, strategies for improving proficiency in competencies, and tools for overcoming barriers [18]. Vignettes and case scenarios are used to reinforce learning.

## Study procedures

We adapted TeamSTEPPS for use with school mental health teams, relying on stakeholder input obtained via a community advisory board. Briefly, core TeamSTEPPS content was largely retained; one module (Situation Monitoring) was de-emphasized, and examples and vignettes were adapted throughout to be more relevant to the school mental health context. See Wolk and colleagues [22] for a more detailed discussion of the adaptation process. We then pilot tested the adapted TeamSTEPPS and examined feasibility and acceptability as well as the impact on team skills and behavior. Data were collected between August 2015 and June 2016.

Participants were 27 individuals (25 team members and 2 leaders) representing 6 school-based teams. Teams typically consist of two masters’ level clinicians supported by several paraprofessional behavioral health workers and case management staff. Per agency leadership’s report, all school-based clinicians had received either in-person or web-based training in CBT through the Beck Community Initiative [7] and were receiving ongoing CBT consultation. No additional inclusion/exclusion criteria were applied.

**Fig. 1:**
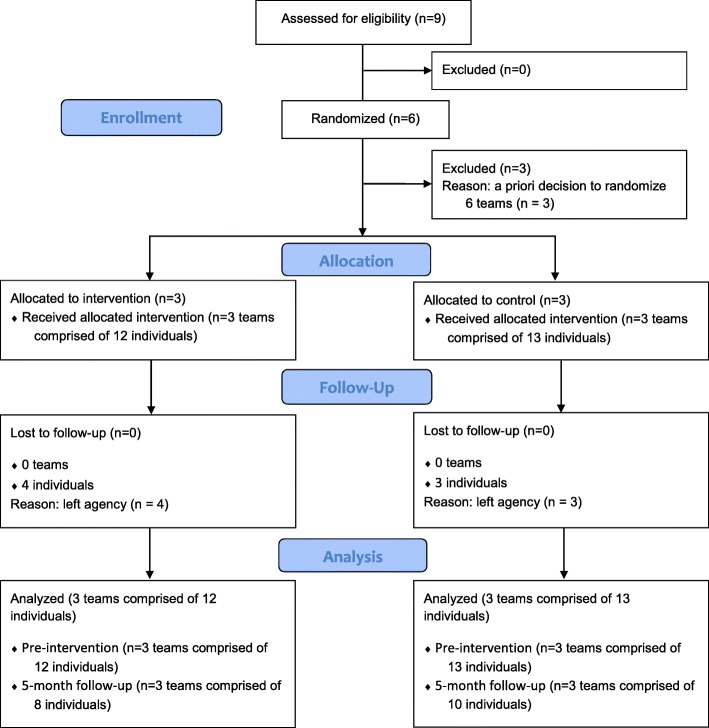
Consort diagram. Consort diagram depicting allocation of teams and attrition of individual participants within teams. Adapted from Eldridge SM, Chan CL, Campbell MJ, Bond CM, Hopewell S, Thabane L, et al. CONSORT 2010 statement: extension to randomized pilot and feasibility trials. BMJ. 2016;355

All study procedures were approved by the relevant Institutional Review Boards as well as the school district’s Office of Research and Evaluation. Informed consent was obtained prior to engagement in research activities. From the pool of nine teams within the agency, three schools and their corresponding teams were randomized to receive the adapted TeamSTEPPS training plus usual support and three schools and their teams were randomized to usual support only (see Fig. 1). Usual support consisted of ongoing weekly group consultation for lead clinicians in CBT implementation through the Beck Community Initiative [7]. Questionnaires were completed post-randomization but prior to training and again at 1 and 5 month post-training. Those who received TeamSTEPPS were also invited to participate in a one-time, in-person, semi-structured interview. Participants were compensated for their time completing study measures and interviews at the rate of $50 per hour.

## Measures

### TeamSTEPPS teamwork perceptions questionnaire

(T-TPQ [23]; administered prior to training and 1 and 5 months post-training). The T-TPQ is a self-report measure of individual perceptions of group-level team skills and behavior. It is based upon the five core components of teamwork that comprise TeamSTEPPS. Each construct is represented by seven questions, totaling 35 items. Items are rated on a five-point scale from “strongly disagree” = 1 to “strongly agree” = 5. A total score is computed by summing all items and higher scores indicate more favorable perceptions. Cronbach’s alpha ranges from 0.88 to 0.95 and convergent validity is adequate [23].

### TeamSTEPPS teamwork attitudes questionnaire

(T-TAQ [24]; administered prior to training and 1 and 5 months post-training). The T-TAQ is a self-report measure of individual attitudes related to the core components of teamwork captured within TeamSTEPPS. Six items measure each of the core teamwork constructs, for a total of 30 items. Items are rated on a five-point scale from “strongly disagree” = 1 to “strongly agree” = 5. A sum score is calculated across items with higher scores indicating more positive attitudes. Constructs exhibit unique variance and Cronbach’s alpha ranges from 0.70 to 0.83 [24].

### Evidence-based practice attitude scale

(EBPAS [25]; administered prior to training). The EBPAS is a 15-item self-report measure of attitudes toward adoption of EBPs. It consists of four subscales: appeal (is EBP intuitively appealing), requirements (would an EBP be used if required), openness (general openness to innovation), and divergence (perceived divergence between EBP and current practice). Items are rated on a five-point scale from “not at all” = 0 to “very great extent” = 4. Higher scores indicate more positive attitudes, with the exception of divergence which is reverse coded. The EBPAS has national norms, demonstrated validity and good internal consistency (subscale alphas range from 0.67–0.91 [26, 27].

### Maslach burnout inventory human services survey

(MBI [28]; administered prior to training and 1 and 5 months post-training). The MBI is a 22 item self-report measure of therapist burnout. Three subscales measure emotional exhaustion, depersonalization, and reduced personal accomplishment. Items are rated on a six-point scale from “never” = 0 to “everyday” = 6 with higher scores on emotional exhaustion and depersonalization and lower scores on personal accomplishment (reverse scored) indicating higher levels of burnout. Satisfactory internal consistency and discriminant and factorial validity have been demonstrated [29–31].

### Qualitative interviews

Individual interviews were conducted with team members (*n* = 7) who engaged in the adapted TeamSTEPPS training approach to elicit views and perspectives about the feasibility and acceptability of the adapted TeamSTEPPS at 1 month post-training. A standardized interview guide consisted of three parts. The first part covered general views about the feasibility of the adapted TeamSTEPPS including perceptions of the (1) extent to which the program can be continued within their organization and (2) extent to which the program can be extended to other school-based mental health teams. The second part queried about acceptability of the adapted TeamSTEPPS, including the extent to which clinicians found the adapted TeamSTEPPS agreeable, palatable, and satisfactory. Finally, in the third section of the interview, we provided respondents with findings from the quantitative data and asked for their reflections.

### Qualitative field notes

At 1 month post-training, the PI took detailed field notes in the 6 schools, including observations of the school building, teams’ therapy and office space, team interactions, and interactions between team members and school personnel.

## Analytic plan

### Quantitative analysis

Mean differences were examined from prior to training to 5 months post-training (i.e., mid school year) and between the intervention and control groups. While participants were clustered within teams, because the variables of interest are individual-level constructs, the unit of analysis is at the individual level.

### Qualitative analysis

Interviews were digitally recorded and transcribed with analyses supported by use of an NVivo database that included field notes. Analysis was guided by an integrated approach [32], which uses an inductive process of iterative coding to identify recurrent themes, categories, and relationships. We identified *a priori* attributes of interest (core components of TeamSTEPPS, acceptability, feasibility) and also used modified grounded theory [33, 34], which provides a systematic and rigorous approach to identifying codes and themes. Using the NVivo qualitative data analysis software program, two members of the research team (CBW and RES) separately coded a sample of 5 transcripts and field notes and compared their application of the coding scheme to assess the reliability and robustness of the coding scheme. Disagreements in coding were resolved through discussion and the codebook was refined. The revised codebook was then applied to all interviews and field notes. CBW coded all transcripts and RS separately coded 62%. Reliability was excellent (*κ* = 0.93).

### Mixed method analyses

Mixed methods were used to compare feasibility and acceptability of TeamSTEPPS, our two key implementation outcomes, among participants who left or were transferred from their team during the course of the school year (i.e., turnover) and those who remained. To integrate the quantitative and qualitative methods, we followed NIH guidelines for best practices [35]. We used findings from the quantitative data to identify patterns in the qualitative interview data. We entered turnover status into NVivo at the individual clinician level and categorized clinicians as those who stayed at their agency or did not. Then, we examined if there were variations in perceptions of acceptability and feasibility between providers that did and did not turnover, which allowed us to identify patterns and make interpretations across these groups based on quantitative categorization.

## Results

### Quantitative

See Table 1 for participant demographics and Table 2 for means and standard deviations of measures by time point. MBI emotional exhaustion scores significantly increased from prior to training (*M* = 2.4, *SD =* 1.1) to 5-month follow-up (*M* = 3.3, *SD =* 1.2). MBI personal accomplishment scores significantly decreased from prior to training (*M* = 5.1, *SD =* .4) to 5-month follow-up (*M* = 4.6, *SD =* .9). The T-TAQ and T-TPQ total scores and MBI depersonalization score did not significantly differ from pretraining to 5-month follow-up. See Table 3.

**Table 1:** Demographic characteristics for intervention and control group participants

	Overall (*n* = 25)	Intervention (*n* = 12)	Control (*n* = 13)
Characteristic	*n* (%)	*n* (%)	*n* (%)
Age, mean years (SD)	36.1 (12.2)	40.8 (13.7)	33.3 (10.8)
Gender			
Male	8 (32.0%)	5 (41.7%)	3 (23.1%)
Female	17 (68.0%)	7 (58.3%)	10 (76.9%)
Race/Ethnicity			
Black or African American	15 (60.0%)	7 (58.3%)	8 (61.5%)
White or Caucasian	4 (16.0%)	1 (8.3%)	3 (23.1%)
Other	4 (16.0%)	2 (16.7%)	2 (15.4%)
Missing	2 (8.0%)	2 (16.7%)	0 (0%)
Professional role			
Clinician	6 (24.0%)	3 (25.0%)	3 (23.1%)
Paraprofessional provider	15 (60.0%)	5 (41.6%)	10 (76.9%)
Case manager	2 (8.0%)	2 (16.7%)	0 (0%)
Missing	2 (8.0%)	2 (16.7%)	0 (0%)
Highest degree			
Master’s	9 (36.0%)	5 (41.7%)	4 (30.8%)
Bachelor’s	13 (52.0%)	4 (33.3%)	9 (69.2%)
Missing	3 (12.0%)	3 (25%)	0 (0%)
Time since degree, mean years (SD)	8.6 (8.4)	9.4 (4.4)	8.3 (9.8)
Turnover			
Left agency during course of the year	12 (48%)	6 (50.0%)	6 (46.2%)

**Table 2:** Means and standard deviations by time point

Measure	Prior to training			5-month follow-up		
	Overall (*n* = 25)	Intervention (*n* = 12)	Control (*n* = 13)	Overall (*n* = 18)	Intervention (*n* = 8)	Control (*n* = 10)
EBPAS total	2.96 (.43)	2.88 (.42)	3.00 (.42)	–	–	–
EBPAS requirements	3.17 (.90)	3.08 (.65)	3.15 (1.12)	–	–	–
EBPAS appeal	3.12 (.63)	3.02 (.74)	3.13 (.50)	–	–	–
EBPAS openness	3.01 (.57)	2.98 (.56)	2.96 (.55)	–	–	–
EBPAS divergence*	2.59 (.72)	2.43 (.71)	2.73 (.77)	–	–	–
MBI emotional exhaustion	2.45 (1.18)	2.58 (1.39)	2.31 (1.05)	3.12 (1.27)	3.44 (1.05)	2.86 (1.42)
MBI depersonalization	.968 (.96)	1.15 (1.12)	.80 (.82)	.90 (1.02)	1.18 (1.40)	.68 (.59)
MBI personal accomplishment	4.75 (.71)	4.68 (.74)	4.80 (.73)	4.64 (.89)	4.25 (.91)	4.95 (.78)
T-TPQ total	107.16 (18.06)	98.64 (16.60)	113.08 (17.17)	105.78 (22.11)	94.25 (23.33)	115.00 (16.96)
T-TPQ team structure	26.04 (4.90)	23.75 (5.28)	28.00 (3.85)	25.33 (7.24)	21.13 (7.88)	28.70 (4.74)
T-TPQ leadership	27.38 (5.84)	26.42 (5.07)	28.00 (6.71)	24.44 (7.94)	20.63 (8.75)	27.50 (6.02)
T-TPQ situation monitoring	25.85 (5.16)	22.83 (4.76)	28.15 (4.08)	26.89 (4.56)	24.63 (5.48)	28.70 (2.79)
T-TPQ mutual support	26.62 (4.74)	24.58 (5.33)	28.15 (3.53)	27.33 (4.98)	25.25 (5.18)	29.00 (4.37)
T-TPQ communication	26.81 (5.25)	26.92 (3.94)	28.92 (5.07)	28.67 (4.49)	27.25 (5.60)	29.80 (3.22)
T-TAQ total	102.29 (11.42)	102.80 (8.27)	105.15 (6.35)	105.65 (8.67)	103.29 (10.22)	107.30 (7.53)
Team structure	25.60 (2.20)	25.17 (2.12)	26.00 (2.27)	25.56 (2.48)	24.50 (2.78)	26.40 (1.96)
Leadership	27.84 (2.27)	27.00 (2.30)	28.62 (2.02)	28.17 (2.15)	26.88 (2.36)	29.2 (1.32)
Situation monitoring	26.00 (2.91)	25.00 (2.30)	26.62 (3.18)	27.28 (2.67)	26.00 (2.92)	28.30 (2.06)
Mutual support	25.04 (3.26)	24.83 (3.10)	24.85 (3.34)	26.00 (3.58)	26.38 (3.07)	25.70 (4.08)
Communication	25.62 (2.93)	25.17 (3.56)	25.69 (2.10)	25.56 (3.47)	25.00 (3.51)	26.00 (3.56)

*reverse scored

**Table 3:** Estimate of differences between pretraining to 5-month post-training scores

Variable	Mean	95% CI
MBI emotional exhaustion	− 0.90	− 1.70 to – 0.11
MBI depersonalization	− 0.16	− 0.69 to 0.36
MBI personal accomplishment	0.49	0.16 to 0.81
T-TPQ total	7.29	− 1.32 to 15.91
T-TAQ total	− 0.80	− 4.55 to 2.95

At pretraining, T-TPQ Total score was significantly different between the control (*M* = 113.1, *SD =* 17.2) and intervention (*M* = 98.6, *SD =* 16.6) groups (see Table 4). At 5-month follow-up, control and intervention teams differed on T-TPQ total score, (*M* = 115.0, *SD =* 17.0 and *M* = 94.3, *SD =* 23.3 respectively). In both instances, the control teams reported more favorable perceptions of teamwork than intervention teams. There were no other significant differences on T-TAQ Total, MBI, and EBPAS scores observed between the control and intervention groups.

**Table 4:** Estimate of differences between intervention and control group scores at pretraining and 5-month follow-up

Variable	Mean	95% CI
Pretraining		
MBI emotional exhaustion	0.28	− 0.73 to 1.29
MBI depersonalization	0.35	− 0.46 to 1.16
MBI personal accomplishment	− 0.12	− 0.73 to 0.48
EBPAS	− 0.12	− 0.46 to 0.23
T-TPQ total	− 14.44	− 28.81 to − 0.07
T-TAQ total	− 2.35	− 8.68 to 3.97
5-month follow-up		
MBI emotional exhaustion	0.59	− 0.69 to 1.87
MBI depersonalization	0.50	− 0.53 to 1.52
MBI personal accomplishment	− 0.70	− 1.54 to 0.14
T-TPQ total	− 20.75	− 40.86 to − 0.64
T-TAQ total	− 4.01	− 13.16 to 5.13

### Qualitative

#### Acceptability of TeamSTEPPS

Participants consistently reported positive perceptions of the adapted TeamSTEPPS program. In particular, participants described appreciating the focus on communication and that the program was evidence-based. Previous experience with team training interventions was limited and consisted primarily of brief facilitated team-building events. The adapted TeamSTEPPS was viewed as appropriate for school mental health teams. One participant noted, “I think it fit really well and I think that was one of the things that stood out for me. I was like, ‘Oh wow, they really know STS (the program name; School Therapeutic Services).’” Challenges to implementation identified included that not all individuals subscribe to the importance of teamwork and that the TeamSTEPPS measures, which had not been adapted, were not optimally worded for use in schools (e.g., contained the word patient instead of student).

#### Feasibility of TeamSTEPPS

Participants described TeamSTEPPS as feasible to implement in the school mental health context in terms of ease of use, burden, and alignment with existing priorities. Specifically, participants reported that the program content was, “fairly easy to follow, I think it’s easy to implement” and “applicable and useful.” The time commitment was described as “very reasonable.” One person stated, “I think it’s just become part of our work day and I don’t think it’s been anything that you feel like it’s a chore because I do think it’s useful and I do think most of the people have seen how it’s useful.” Participants suggested that the program was most likely to be maintained with support from clinical managers, agency leadership and by integrating TeamSTEPPS with the agency’s larger agenda. Participants varied in their perceptions of leadership’s support for TeamSTEPPS implementation. Some participants reported that they already had support from managers and leaders to implement TeamSTEPPS while others remarked that current supports were not optimal.

#### Team structure

When asked to describe the composition of their team, the clear majority of STS team members reported that their team consisted of a clinical manager, clinicians, and paraprofessional behavioral health workers. Less frequently, identified team members also included agency leadership (e.g., clinical director) and school personnel (e.g., school counselor, principal, teachers). Turnover, transfer of personnel to different schools/teams, and relying on part-time or seasonal staff were noted to hinder teamwork.

#### Leadership

Team leaders were overwhelmingly new to their assigned school that year, having been promoted from within the agency. The description of leadership was polarized: either a leader actively worked to enhance teamwork (e.g., increasing the frequency of meetings and communication between staff, being attentive to feedback, holding staff accountable) or a leader hindered or did not support effective teamwork by, for example, frequently being off site or dismissing staff’s questions or concerns. The importance of having a strong leader to a team’s success was frequently noted.

#### Communication

Individuals spoke frequently about the importance of communication among team members and between teams and school personnel. Team members spoke about good communication being particularly important when managing challenging clinical cases. Also important was being able to communicate openly about frustrations or burnout. Poor communication was acknowledged to hinder success. Team members also noted the value of having formalized meetings and operationalized procedures for communicating. Observed meetings were reportedly brief (less than 10 min) and described as typically including a discussion of how the team members were feeling that morning, administrative matters (e.g., reminders to submit notes on time), staffing assignments, and discussions of treatment plans. There was great variability in whether meetings were conducted collaboratively or run by the manager, who provided updates and information to the staff. Respondents frequently highlighted having good communication between teams and schools.

#### Mutual support

Participants spoke about supporting one another in their personal lives and in their jobs. Individuals frequently spoke about the importance of the relationships they had formed with their team members, the importance of offering support, and being able to rely on their teammates to go above and beyond to help them meet deadlines. Less frequently, staff expressed frustration about the support they needed to provide their team (e.g., “I’m doing the work of three people in a day.”).

#### Situation monitoring

Situation monitoring was infrequently noted or observed, and only occasionally did staff talk about the importance of monitoring team members. One person stated, “I think the team works well in identifying who has good relationships with different children and we can kind of see who’s stressed out; if a child’s like taking certain staff to a limit I might step in and say, ‘child, come to my office for a little bit’ and we’ll just sit down and just talk.” Another noted, “When someone comes in and they look frustrated that day and maybe you don’t put them with the [most challenging] child first thing in the morning.”

#### Barriers and facilitators

Reported and observed barriers included being in under-resourced schools, having limited financial resources for the program, getting necessary information in a timely manner, staff being resistant to change/inflexibility, staff being uninterested in improving their team’s relationship, being split between multiple schools, frequent turnover and transition of staff between schools, reliance on part-time and contracted staff, limited time/being overextended, frequent distractions, lack of accountability, and the limited training of some staff. Reported facilitators included having a strong leader who values teamwork, staff who often do whatever they can to help students, having staff who pride themselves in their work, and having developed collaborative relationships with school leadership.

#### Suggested modification to TeamSTEPPS

The most frequent suggestions for further adaptations to the program were to provide (1) regular booster trainings in TeamSTEPPS principles to new and existing staff and (2) in-school coaching or implementation support to teams. Less frequently, participants suggested using incentives, leveraging competition/peer comparison, conducting “micro” trainings with one school team at a time, and including more “real-world” examples drawn from the agency/schools in training. Also noted was the importance of agency leaders showing that they were behind the program. Suggestions for accomplishing this included having agency leaders become TeamSTEPPS trainers and integrating TeamSTEPPS into the “framework of the program.”

#### Clinical skills and strategies

Participants frequently spoke about their work being trauma-informed, strength-based, and following sanctuary principles [36]. Clinical leaders expressed frustrations with what they perceived as a lack of training in evidence-based practices among other clinicians or paraprofessional staff. Observed clinical strategies included use of token economies and reinforcement, supportive counseling, art therapy, and directive problem solving. Teams frequently were observed playing games and completing art projects of a non-therapeutic nature with students. Punishment was threatened or used at times, for example, warning a child that if they did not behave, the clinician would call his caregiver.

#### Culture

When asked to describe the culture of his/her team participants largely described a positive team culture. One participant stated, “I feel like everybody does truly have the best interest of the child at heart and I can say…from a behavior standpoint it is a very difficult school. But I see staff not only working with kids on behavior things, but even some academic help. [It] is really outside their role but…you don’t get people saying, ‘Well I don’t do that. This child needs help. I’m going to help this child.’ So I would say everybody really has that ‘want to be helpful’ spirit.” Others spoke of being in a “team-based organization.” Less frequently, participants noted that one or more members of their team had attitudes toward teamwork that were less than optimal and that this negatively affected team culture. Collaboration and collegiality were cited as important to the culture within a team, and the impact of how collaborative the school and STS were with one another was also noted to impact team culture.

#### Physical environment

While kindergarten-through-5th-grade schools were observed to be relatively calm and orderly, the K-8 schools were noticeably louder, more chaotic, and had more instances of adults raising their voices and student altercations. Visual displays in STS rooms frequently included efforts to track students and staff assignments as well as messaging related to sanctuary principals [36]. Some STS teams had two rooms in the school (typically one used as a staff office and another for private meetings/therapy), while others only had access to a single space to be used for office/administrative tasks and therapeutic activities. In these schools, staff often reported using hallways, auditoriums, or other space in the schools for therapy.

### Mixed methods

Differences in feasibility and acceptability, our two key implementation outcomes, were examined among staff who did and did not leave the team during the course of the school year. Qualitative interviews of feasibility and acceptability were completed with staff while they were still employed on the school-based team (i.e., prior to their resignation or leaving the school). Regarding feasibility, those who did not turnover more often discussed feeling support for implementing TeamSTEPPS from agency leadership and their team’s manager and described concrete changes that had been made since the training (e.g., “[manager] has been really good about popping by and checking in on people throughout the day” and “we’ve been using [staff] feedback to adjust scheduling”). They described specific strategies from the training, such as conflict resolution techniques, as helpful and expressed confidence in their ability to continue to implement the strategies. Those who did turnover, by contrast, were more likely to say they needed agency leadership and team managers to identify TeamSTEPPS as a priority in order for changes within the team to be made, to identify needing further training and support in TeamSTEPPS, and to cite competing priorities as barriers to implementation.

Regardless of turnover, team members generally described TeamSTEPPS as highly acceptable. Those who did not turnover described specific components of TeamSTEPPS they liked (e.g., communication strategies, debriefing meetings) while those who did turnover tended to provide examples less relevant to key TeamSTEPPS content (e.g., enjoying the icebreaker activity) or to suggest that providing incentives for participation would make TeamSTEPPS more acceptable.

## Discussion

In this pilot study, we adapted TeamSTEPPS for school mental health teams and demonstrated feasibility and acceptability with co-located school mental health teams implementing CBT in urban, under-resourced schools. TeamSTEPPS dimensions were highly relevant in this context and the domains of communication and leadership were particularly salient to participants. The intervention did not lead to significant improvements in team skills and behaviors or provider burnout; however, the lack of significant quantitative findings may be related to the small sample size. An examination of themes from qualitative interviews identified important areas of perceived impact and suggested that additional modifications to TeamSTEPPS may be needed to enhance the impact on school-based mental team processes.

While teamwork perceptions and attitudes were not impacted by the training, provider burnout significantly increased from prior to training to 5-month follow-up. While contrary to expectations, the results are not surprising given that our sample consisted of school-based providers where pretraining surveys took place during summer break and follow-up data collection occurred midway through the school year when burnout and stress on teams may have been higher due to secular considerations. Future studies should investigate whether teamwork perceptions and attitudes are modifiable factors that can reduce provider burnout which is important in such a high turnover context.

The importance of communication and a strong team leader were frequently highlighted by qualitative interview participants. This is consistent with the literature highlighting leadership as a key factor in implementation science frameworks [37]. It may be especially critical in the often chaotic and challenging environment of under-resourced schools [38]. By contrast, frequent staff turnover and challenges in the school mental health team relationship emerged as barriers. Additional supports to implement TeamSTEPPS, including regular booster training to address staff turnover and ongoing consultation, were suggested by interview participants to maximize the impact on team processes. These suggested that adaptations highlight the value of conducting formative qualitative work when extending an intervention to a new context [39]. Organizational factors have been shown to predict employee turnover [40], further underscoring the importance of attending to contextual factors.

Given the high level of turnover, we used mixed methods to examine differences in feasibility and acceptability (our two key implementation outcomes) among staff who did and did not leave the team during the course of the school year. Regarding feasibility, our results suggest that those who left their teams may have felt less support from leadership and may have taken less personal responsibility for the functioning of their team and the implementation of TeamSTEPPS. It is possible that leadership support may directly impact one’s tenure on the team, though we were unable to examine that specifically in the present study. Regardless of turnover, team members generally described TeamSTEPPS as highly acceptable, supporting the promise for this intervention with school-based mental health teams.

These preliminary results suggest that teams liked TeamSTEPPS because they believed it was relevant to their context, provided important content (especially around communication strategies), was evidence-based, and mostly feasible to implement in terms of time burden and training expectations. However, staff turnover presented a significant barrier to changing team skills and behavior. In addition to high turnover, the study is limited by a small sample size and that we partnered with providers from a single agency in a single school district. This may limit generalizability. Future studies should draw from larger samples and multiple organizations. Our data also indicated that additional modifications to TeamSTEPPS for this context are needed prior to wider deployment. In particular, a mechanism for ensuring ongoing training and support for teams implementing TeamSTEPPS will be important given high turnover. Contextual factors likely vary considerably across schools, underscoring the need for formative mixed methods research like this utilizing an implementation lens to account for the local drivers of success. Given the importance of context, we also think that there is a need to engage school personnel directly in further adapting and implementing TeamSTEPPS. To date, we have only partnered with mental health providers in the adaption process. Working with all relevant stakeholders to further refine TeamSTEPPS is important to ensure both acceptability and feasibility of the intervention as well as the development of appropriate implementation supports.

## Conclusions

Results suggest that TeamSTEPPS is a promising approach but that further adaptations are needed to improve fit for school mental health teams. This project informs our understanding of strategies to improve evidence-based practice implementation among school-based mental health care teams and beyond, as team-based models of care are increasingly being utilized in other mental health settings.

## Data Availability

The datasets used and/or analyzed in the current study are available from the corresponding author on reasonable request.

## Abbreviations

EBPAS: Evidence-Based Practice Attitude Scale
MBI: Maslach Burnout Inventory Human Services Survey
STS: School therapeutic services
TeamSTEPPS: Team Strategies and Tools to Enhance Performance and Patient Safety
T-TAQ: TeamSTEPPS Teamwork Attitudes Questionnaire
T-TPQ: TeamSTEPPS Teamwork Perceptions Questionnaire
